# DNA transposon-based gene vehicles - scenes from an evolutionary drive

**DOI:** 10.1186/1423-0127-20-92

**Published:** 2013-12-09

**Authors:** Kristian Alsbjerg Skipper, Peter Refsing Andersen, Nynne Sharma, Jacob Giehm Mikkelsen

**Affiliations:** 1 Department of Biomedicine, Aarhus University, Wilh. Meyers Allé 4, DK-8000, Aarhus C, Denmark; 2 Present address: IMBA - Institute of Molecular Biotechnology GmbH, 1030, Vienna, Austria

## Abstract

DNA transposons are primitive genetic elements which have colonized living organisms from plants to bacteria and mammals. Through evolution such parasitic elements have shaped their host genomes by replicating and relocating between chromosomal loci in processes catalyzed by the transposase proteins encoded by the elements themselves. DNA transposable elements are constantly adapting to life in the genome, and self-suppressive regulation as well as defensive host mechanisms may assist in buffering ‘cut-and-paste’ DNA mobilization until accumulating mutations will eventually restrict events of transposition. With the reconstructed *Sleeping Beauty* DNA transposon as a powerful engine, a growing list of transposable elements with activity in human cells have moved into biomedical experimentation and preclinical therapy as versatile vehicles for delivery and genomic insertion of transgenes. In this review, we aim to link the mechanisms that drive transposon evolution with the realities and potential challenges we are facing when adapting DNA transposons for gene transfer. We argue that DNA transposon-derived vectors may carry inherent, and potentially limiting, traits of their mother elements. By understanding in detail the evolutionary journey of transposons, from host colonization to element multiplication and inactivation, we may better exploit the potential of distinct transposable elements. Hence, parallel efforts to investigate and develop distinct, but potent, transposon-based vector systems will benefit the broad applications of gene transfer. Insight and clever optimization have shaped new DNA transposon vectors, which recently debuted in the first DNA transposon-based clinical trial. Learning from an evolutionary drive may help us create gene vehicles that are safer, more efficient, and less prone for suppression and inactivation.

## Review

### Introduction

Since its birth the field of therapeutic gene transfer has travelled on a rough road from initial optimism through hype and disappointment towards scientifically well-founded hope and signs of clinical applicability and success. Indeed, incipient confidence is fuelled by promising preclinical and clinical findings and by an increasing wealth of improved gene transfer technologies. Continuous focus on gene carriers and their biological properties, immune responses, and interactions with host cells already has provided clinical benefit [1-3] and will continue to do so. As one of many recent examples, our improved understanding of determinants that control viral gene insertion – being the result of the combined efforts of virologists and gene therapists - not only explains us why integrating viral vectors tend to insert near and deregulate cellular genes but is likely also to pave the way for vector systems with altered integration profiles.

With the revived *Sleeping Beauty* DNA transposon in the driver’s seat, transposable DNA elements have emerged as promising nonviral vehicles for persistent gene delivery [4,5]. The simple gene integration machinery of cut-and-paste transposons provides non-viral gene delivery systems – naked plasmid-based DNA vectors as the most primitive system – with the ability to insert transgenes into target cell genomes. Nonviral vector types that have traditionally suffered from short-term expression of transgenes now facilitate long-term therapeutic levels of gene expression due to active genomic insertion of the transgene. Potency of transposon-based vectors in preclinical settings has been demonstrated in liver [5-10], lung [11-16], skin [17], and brain [18,19] of adult mice injected with plasmid DNA. Packaged in synthetic wrappings consisting of cationic polylysines or hydrophobic lipids, transposon vectors may eventually mimic integrating viral vectors, but in a format that is potentially less toxic and less immunogenic than viral vectors and which is well-suited for large-scale vector production.

Mobilizing nucleic acids is the key to every application of gene transfer. This is why gene therapists look for inspiration in families of viruses which have through evolution adapted to transport genetic cargo and in some cases to pick up and mobilize cellular genes. In case of transposable elements, with or without viral origin, mammalian genomes provide the inspiration. The mobility of nucleic acid sequences in discrete genetic segments has incredible impact on the dynamics and evolution of almost any genome. Driving their survival, transposable elements possess a unique ability to multiply within genomes, leading to an abundance of genetic entities with no obvious beneficial effects on the host. These small mobile units are genetic parasites, which have adapted to the gene expression machinery of the host and each typically encodes one or two proteins that catalyze their continuous spread within a genome potentially setting the stage for horizontal transmission.

Transposable elements are remarkably widespread and appear to have colonized almost every living organism. In humans, about four million transposable elements, most of them inactive fossil remnants of once actively transposed sequences, represent a stunning almost 50% of the entire genome [20-22]. With such abundance the mobile elements, active or inactive, will inevitably affect the overall ecology of the genome. Indeed, mutations caused by transposon insertion or by chromosomal recombination between inserted transposable elements may serve to increase genetic variation as a platform for selection and evolutionary change. Hence, the activity of transposons may impact and/or favor the adaptability and evolution of species. Examples of specific insertions having beneficial effects on their hosts are rather few, and the accumulation of mobile elements is in general expected to affect host fitness in a negative direction. In smaller multicellular eukaryotes, like fruit flies, fitness is inversely correlated with transposable element copy number [23-25]. Such elements, in the past referred to as *selfish* or *self-promoting* elements, may thus multiply to an extent that is defined and limited by the natural selection against their carriers.

The co-existence of a parasitic mobile element with its host relies on the element possessing an evolutionary stable level of activity. Too active elements may simply replicate too efficiently, resulting in numerous insertions that are harmful to the host and eventually fuel extinction of both host and element. As an expected consequence, transposition may be regulated by self-suppressive mechanisms by the element itself and by defensive host mechanisms. These adaptations that may differ from element to element and from host to host collectively define the functional and biological properties of a transposable element.

Since Yant and co-workers in a pioneering study demonstrated *in vivo* potency of *Sleeping Beauty* DNA transposon-based gene delivery to the liver of adult mice, continued efforts have been made to understand and further develop DNA transposon elements as gene carriers in mammalian cells and animals. As efforts to optimize and employ *Sleeping Beauty* vectors have become more and more frequent, the battery of transposable gene vehicles with activity in mammalian cells and with relevance for gene transfer in humans is rapidly expanding (see Table 1 for overview and specific features). In addition to the *Sleeping Beauty* transposon which is derived from the genome of white cloud mountain minnow (*Tanichthys albonubes*) [4], the *piggyBac* element, isolated from the cabbage looper moth *Trichoplusia ni *[26,27], has shown high levels of DNA transposition in human cells [28,29]. In addition, *Frog Prince* derived from the genome of the leopard frog *Rana pipiens *[30], *Himar1* derived from the hornfly *Haematobia irritans *[31], *Tol2* isolated from the genome of the Japanese medaka fish *Oryzias latipes *[32], and *Passport* derived from the flatfish *Pleuronectes platessa *[33] transpose in mammalian cells. Also, the ancient human *Hsmar1* transposon is efficiently mobilized in human cells by a reconstructed ancestral *Hsmar* transposase [34]. Recently described elements with robust mobilization in human cells include *piggyBat* isolated from the bat *Myotis lucifungus *[35] and *TcBuster* from the red flour beetle *Tribolium castaneum *[36,37]. Common to this growing collection of mobile elements is that transposition appears not to rely on species-specific host factors. Still, host factors like DNA-bending proteins may support the transposition process, as shown for *Sleeping Beauty* and *Frog Prince *[38]. As a result, some cell types are easier stably transfected with DNA transposon vectors than others [39]. Accordingly, vector systems based on the distinct elements may be favoured in different cell types making a strong argument that parallel efforts to investigate and develop distinct, but potent, systems will benefit the broad applications of transposon-based gene transfer. However, we do not seek here to review the entire package of biological properties that make transposons like *Sleeping Beauty* and *piggyBac* ideal for nonviral gene integration purposes in mammalian cells. Numerous excellent reviews already tell that story [40-49]. Instead, with examples from the distinct families of DNA transposable elements we try here to make direct connections between the mechanisms that drive transposon evolution and some of the challenges in transposon-based gene transfer. Experience with *Sleeping Beauty* in particular tells us that transposon vehicles are travelling with evolution as a rear-seat passenger. By understanding in detail the evolutionary journey of transposons, from host colonization to element multiplication and inactivation, we may be better prepared for utilizing and optimizing transposon-based gene transfer. We argue here that early generations of DNA transposon-derived vectors may suffer from inherent traits of their mother elements, but that new carefully engineered vector generations will address - and in some cases have addressed - key issues rendering transposon gene vehicles safer, more efficient, and less prone for suppression and inactivation.

**Table 1:** DNA transposon vectors and their main characteristics

**Transposon**	**Transposon family**	**Target sequence**	**Integration preference**	**Cargo capacity**	**Footprint**	**Overproduction inhibition**	**Hyperactive transposase**	**References**
*Sleeping Beauty*	Tc1/*mariner*	TA	Fairly random (31-39% into genes)	~ 10 kb	C(A/T)GTA	Yes	SB100X	[50-54]
*Frog Prince*	Tc1/*mariner*	TA	ND	ND	C(A/T)GTA	ND	None	[30]
*Hsmar1*	Tc1/*mariner*	TA	Fairly random (44% into genes)	ND	T(T/A)A	Yes	None	[34,55,56]
*Himar1*	Tc1/*mariner*	TA	Fairly random	4 kb	ACTA	Yes	C9	[31,57-59]
*Passport*	Tc1/*mariner*	TA	Transcriptional units (63% into genes)	ND	ND	Yes	None	[33]
*Tol2*	*hAT*	8 bp random sequence	Transcriptional units (39-48% into genes)	> 11 kb	8 bp random sequence	Limited	None	[60-62]
*TcBuster*	*hAT*	8 bp random sequence	Transcriptional units	ND	8 bp random sequence	ND	TcBuster_CO_V_596_A	[36,37]
*PiggyBac*	*PiggyBac*	TTAA	Transcriptional units (47-67% into genes)	100 kb	None	Conflicting reports	7pB, hyPBase	[27-29,63,64]
*PiggyBat*	*PiggyBac*	TTAA	Transcriptional units	ND	ND	ND	None	[35]

ND: Not determined.

### DNA transposable elements – a zoo of endogenous parasites

The far majority of transposable elements in mammalian genomes replicate essentially like retroviruses (except for the lack of an extracellular phase). These so-called *retrotransposons* replicate through a copy-and-paste mechanism that involves production of an RNA intermediate transcribed from the donor element. A reverse-transcribed DNA version of the element is re-inserted elsewhere in the genome. Such retrotransposons include (i) long interspersed nucleotide elements (LINEs) with two open reading frames and an internal promoter, (ii) short interspersed elements (SINEs) that are parasitic on the LINE replication machinery as they have none of their own, and (iii) LTR retrotransposons which resemble retroviruses in structure with *gag* and *pol* genes flanked by long terminal repeats [65,66].

DNA transposable elements that move around the genome by a cut-and-paste mechanism are usually shorter than retrotransposable elements, typically in the range from 1 to 5 kb, and have terminal inverted repeats (IRs) that contain binding sites for the transposase, which is often the only protein encoded by the transposon. The IRs of different elements have variable lengths but may in some cases be more than 700 bp long [67]). Upon transposase complex formation facilitated by binding of transposase subunits to the IRs, the element is cut out of the genome and inserted elsewhere in the genome, in some cases with preference for sites in the immediate neighborhood of the original donor locus. As opposed to retrotransposons, DNA transposons leave behind none or only a short footprint consisting of a few nucleotides that are copied during integration and a few nucleotides that are inserted during DNA repair after element departure.

**Figure 1:**
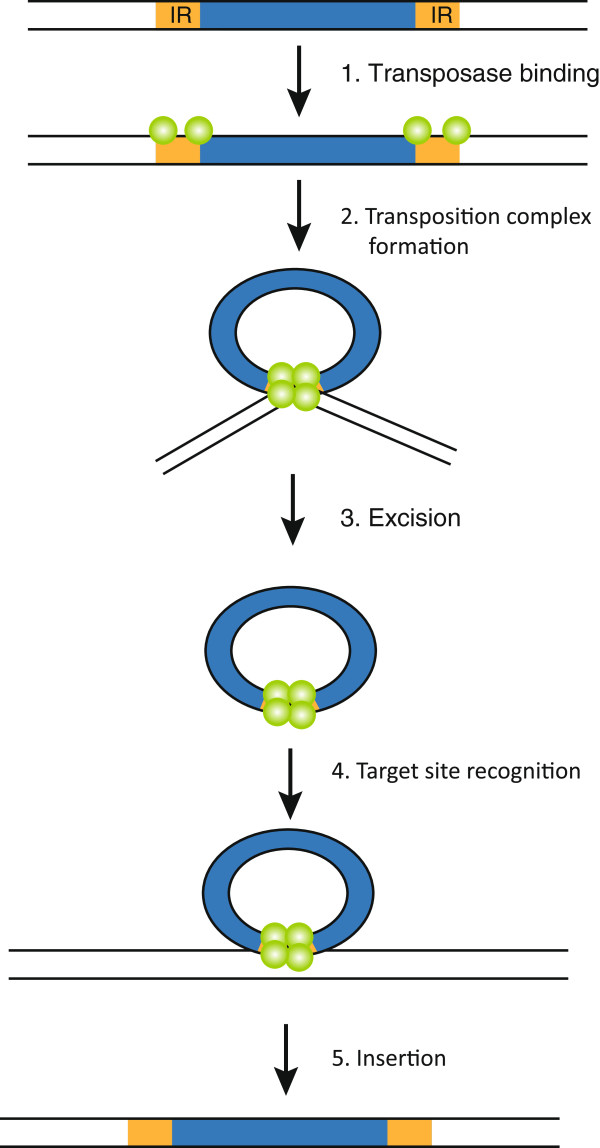
**Model of cut-and-paste transposition.** Transposase proteins (green spheres) recognize the terminal inverted repeats (IRs, orange boxes) and form a circular pre-excision synaptic complex from which the transposon is excised. Formation of the synaptic complex, allowing close association of the two transposon ends, involves the formation of transposase tetramers [74]. Subsequently, the transposition complex recognises a target site and the transposase proteins mediate integration of the transposon. The figure shows the proposed mechanisms for Tc1/mariner-type (e.g. *Sleeping Beauty*) DNA transposition. Yellow boxes marked with IR represent the terminal inverted repeats.

**Figure 2:**
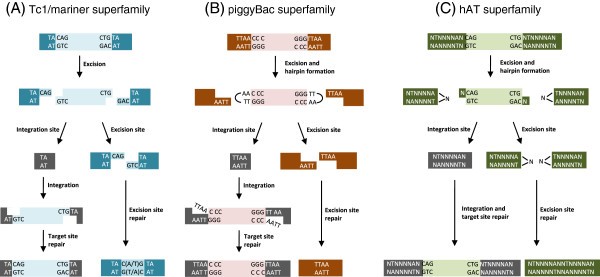
**Schematic representation of cut-and-paste transposition. (A)** Transposition of Tc1/*mariner* elements (like *Sleeping Beauty* and *Frog Prince*) leads to double-stranded breaks and formation of a 2 or 3 bp 3′-overhang at the excision site (a 3 bp overhang is shown). DNA repair by host-encoded enzymes creates a characteristic footprint at the excision site. Integration occurs at TA dinucleotides which are duplicated upon transposition. The single-stranded gaps are repaired by host-encoded enzymes. **(B)***PiggyBac*-mediated excision is followed by hairpin-formation at the transposon ends. After integration into TTAA target sites that are consequently duplicated, the single-stranded breaks are repaired by ligation. The 5′ TTAA overhangs created at the excision site anneal, thus repairing the double-stranded break without leaving any footprint. **(C)***hAT* transposition creates hairpins at the ends of the flanking donor DNA. Integration is targeted to NTNNNNAN target sites, and as with Tc1/*mariner* and *piggyBac* families, *hAT*-mediated integration creates target-site duplication. Excision site repair leaves a random footprint.

Cut-and-paste transposons are defined by their similar structure and mechanism of transposition. All elements are composed of a central transposase-coding region flanked by terminal IRs. Cut-and-paste transposition, first demonstrated for *P* elements and Tc1/*mariner* elements [68,69], is catalyzed by the transposase protein, facilitating transesterification and transposon excision after binding to the IRs (reviewed in [70] and [55]). The transposase-bound excised transposon is then inserted elsewhere in the genome (Figure 1). While the overall cut-and-paste mechanism is shared by all the superfamilies, some of the molecular details of the mechanism may vary between elements (see Figure 2 for an overview of transposition by Tc1/*mariner piggybac*, and *hAT* transposons). In Tc1/*mariners*, for example, the element is excised by transposase-mediated double-strand breaks, leaving 2 or 3 bp 3′-overhangs at the transposon ends [69,71]. After excision, the element is inserted elsewhere into at TA-dinucleotide target-site [50,69], and repair of the 3′-overhang generated during excision creates, together with the target site duplication (TSD), a characteristic transposition footprint. In the *piggyBac* superfamily, in contrast, excision of the element results in hairpin formations at the excised transposon ends, and the 5′-TTAA overhangs created in the flanking DNA after excision anneal in the absence of DNA synthesis, leaving an intact excision site without any transposition footprint [72]. Also the *hAT* transposons (like *Tol2*) form hairpin structures during transposition, but in this superfamily the hairpins are formed at the ends of the flanking donor DNA instead of at the ends of the excised element [73].

The family of mobile DNA elements also include non-autonomous Miniature inverted-repeat transposable elements (MITEs), whose transposition relies on an *in trans* supply of transposase protein [75]. Sharing the terminal repeat sequences with those of DNA transposons, MITEs have likely evolved from autonomous elements. The exception to the rule among DNA transposons is the Helitron family of rolling-circle (RC) DNA transposons [76,77]. These atypical elements transpose by a replicative mechanism that they share with circular ssDNA bacteriophages, bacterial plasmids, and geminiviruses.

### The evolutionary journey of cut-and-paste DNA transposons

With common mechanisms of transposition and supposedly similar modes of regulation cut-and-paste DNA transposons embark on an evolutionary journey. Following invasion of a new host the newly arrived DNA transposon must proliferate and spread in the host population. After establishment and initial spreading, however, host- and self-regulation starts limiting transposition and inactivating transposons, eventually hampering mobilization of the element in the host. Without being able to transpose and proliferate, the inactive transposons are left as relics in the host genome and may eventually be lost by genetic drift.

To persist and continue their evolutionary path mobile transposons must colonize new hosts. The transmission of transposons between species, known as horizontal transfer (for excellent review see [78]), has been documented for most types of transposable elements. Most of the reported putative horizontal transfer events, however, involve cut-and-paste DNA transposons, supporting the notion of horizontal transfer being essential for DNA transposon survival [79-82]. Indeed it has been suggested that the cessation of DNA transposon activity in anthropoids around 37 million years ago was caused by sudden inability to perform horizontal transfer to these hosts [83]. Though *P* elements and Tc1/*mariners* have spawned the strongest and most numerous horizontal transfer documentations (e.g. [84] and [85]), horizontal transfer of elements from other DNA transposon superfamilies has been reported, including *Tol2 *[86] of the *hAT* superfamily and *piggyBac *[87], the latter which was discovered on the basis of horizontal transfer events [88]. Despite the numerous studies trying to identify the vectors that transport mobile DNA elements between species, these remain elusive. Suffering from the lack of clear evidence, viruses [89,90] and various parasites [91,92] have been suggested as carriers of DNA transposons. For successful colonization of a new host the transposon not only needs to transfer to a new host, but needs also to proliferate and spread in the new genome to establish a population [93]. Obviously, the colonizing element is during this phase faced with the challenge of successful replication.

### Models of replicative transposition

#### *Replication by transposition from replicated to unreplicated DNA*

**Figure 3:**
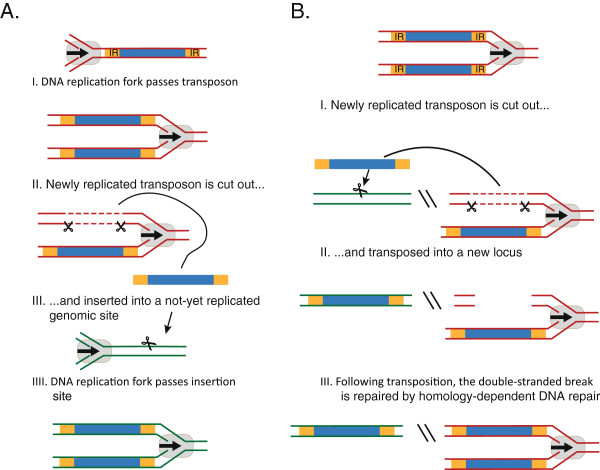
**Models of replicative transposition. (A)** After replication, the transposon is excised and integrated into a yet unreplicated genomic site thus duplicating the newly inserted transposon. **(B)** The double-stranded break created by transposition at newly replicated DNA is repaired using the sister chromatid as a template for homology-directed DNA repair, leading to reconstitution of the excised transposon. IR, terminal inverted repeat.

In consideration of the abundance of DNA transposons and the cut-and-paste mechanism of transposition we are faced with an obvious question: how can elements, which move in a non-replicative way, ever increase in numbers within a genome? Based on genetic studies of the *Activator/Dissociation* (*Ac/Ds*) DNA transposable elements in maize, Chen *et al.* suggested a replicative mode of transposition [94]. According to this model the transposon is replicated during DNA replication, when it transposes from an already replicated site in the genome to a yet unreplicated site (Figure 3A). The model implies a preference for transposition immediately after passage of the replication fork, which has been suggested to rely on DNA methylation [95,96]. Specifically, in a series of methylation studies on *Ac/Ds* transposition [95], Ros and Kunze showed that full CpG methylation of the *Ac/Ds* transposon severely inhibited transposition in a way that could be overcome by DNA replication. They revealed by *in vitro* studies of transposase binding to differently methylated *Ac/Ds* fragments that the effect of DNA replication relied on selective binding of the transposase to hemimethylated transposase binding sites. In fact, this selectivity was even shown to be specific for one of the two hemimethylated daughter elements produced by replication, making this element over six times as active. Though further mechanistic substantiation is needed, these data, supported by earlier studies on *Ac/Ds* transposition [94,96,97] and prokaryotic cut-and-paste transposons [97-100], provide an attractive model for replicative transposition of eukaryotic cut-and-paste transposons.

Additional support for replication of transposons through jumping to unreplicated DNA is provided by early studies on prokaryotic cut-and-paste transposons. Results from the prokaryotic DNA transposons *Tn10/IS10 *[98] and *Tn5/IS50 *[97,99] of the *IS4* family [100] reveal transpositional regulation very similar to that found for the eukaryotic *Ac/Ds* elements. Hence, both elements transpose preferentially from hemimethylated DNA with a specific preference for one of the strands. Interestingly, these two elements are, unlike many other prokaryotic DNA transposons, restricted to non-replicative cut-and-paste transposition [101,102]. This also gives reason to the absence of *dam* (DNA adenine methylase) sites in other prokaryotic DNA transposons like *IS1* and *IS26 *[98,103,104]. However, these elements do not need to tie their transposition to the passage of the replication fork as they can replicate by alternative mechanisms.

#### Replication by homology-dependent DNA repair

Another model for replication of transposable elements arose from studies by Engels *et al.* of homolog-dependent high-frequency loss of the *P* element DNA transposon in *Drosophila *[105]. The model is based on the formation of a double-stranded DNA break (DSB) at the site of transposon excision and subsequent repair by a homologous DNA template (Figure 3B). If the template is the homologous chromosome (assumed to carry the wild-type sequence at the insertion point) the transposon will be lost, as observed by Engels *et al*. However, if the template is the sister chromatid, which was shown to be preferred in a study of *P* elements [106], the transposon will be restored at the excision site, and the transposition is then replicative. Similar results have been obtained in studies of the nematode element Tc1 of the Tc1/*mariner* superfamily, indicating that this model could be universal [107,108].

**Figure 4:**
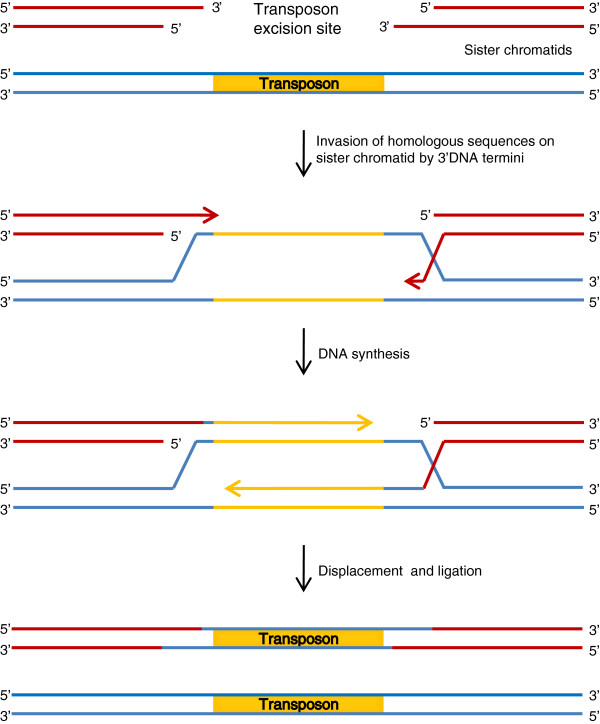
**Homology-dependent DNA repair by synthesis-dependent strand annealing (SDSA).** Following excision, the 3′-DNA termini from the double-stranded breaks invade the homologous sequence on the sister chromatid. The homologous sequence is then used as template for DNA synthesis and the final, elongated 3′-DNA termini anneal to each other and are joined with the 5′ ends by ligation.

Synthesis-dependent strand annealing (SDSA, first described for the T4 phage [109]) has been suggested to be the molecular mechanism underlying this homolog-dependent gap repair [110]. According to this mechanism, 3′-DNA termini left at the DSB independently invade a double-stranded homologous sequence, extend by DNA synthesis using the homologous sequence as template,and after displacement anneal to each other in a region of overlap (Figure 4). Hereafter non-overlapping sequence is removed, and remaining nicks are sealed by ligation. Unlike events of homologous recombination (HR), repair by SDSA does not require cross-overs, and the homologous template is simply copied into the DSB, thus explaining the high deletion and duplication rates observed [105,107]. The SDSA pathway has also been suggested to account for the creation of the non-autonomous *Ac/Ds* elements by incomplete repair after transposon excision [111], indicating that this model might also apply to the *hAT* superfamily of cut-and-paste DNA transposons.

The bigger picture of repair after transposon excision also involves other mechanisms. In non-homologous end-joining (NHEJ; see [112] for recent review), the broken ends in the DSB are joined, resulting in a characteristic footprint (consisting of the overhang sequence flanked by target site duplications) revealing that a transposon was once there. Studies of the repair products of both the *P* element [113] and the reconstructed Tc1/*mariner Sleeping Beauty* element [9,114] indicate that NHEJ is a major pathway of DSB repair after transposon excision. Studies on *P* elements indicated interaction between the *P* element inverted repeats and the Drosophila homolog of the mammalian protein Ku70 [115] (part of DNA-PK, a main component in NHEJ). Later results revealed direct interactions between the *Sleeping Beauty* transposase and Ku70 [114], suggesting a role of the transposase in recruiting the DNA-binding Ku70/Ku80 heterodimer subunit of DNA-PK to the DSB to promote NHEJ and ensure genomic stability. Knocking out Ku, however, did not abolish DSB repair. Instead, analysis of the DSB repair products indicated that the homology-dependent SDSA repair pathway was now in place for repair [114].

Studies in *Drosophila* as the host of *P* elements reveal a complex scenario in which different mechanisms - NHEJ, SDSA and yet another mechanism referred to as single-strand annealing (SSA) - are major competing or complementary pathways [116-119]) for DSB repair. Factors like genomic context [117], cell cycle phase [119] and developmental stage [118] may determine which pathway is used. Nevertheless, transposons have found ways to influence the process either by directly interacting with factors of the repair pathways, as observed for *Sleeping Beauty* transposase-Ku70-interaction [114], or by modulating the relevant host factors, as has recently been found to be the case for *Sleeping Beauty *[120]. Here it was found that the *Sleeping Beauty* transposase halts cell cycle progression from G_1_ through interaction with the transcription factor Miz-1 (which regulates several genes involved in cell cycle regulation [121-123], including D1 [124]). Hereby *Sleeping Beauty* prolongs the G_1_-phase, possibly favoring transposition in this phase during which NHEJ seems to be favored over homolog-dependent repair [125,126]. The complex balance of competing repair mechanisms probably reflects basic evolutionary selection parameters such as transposon replication (by SDSA), genomic stability (e.g. by avoiding uncontrolled transposon replication), and long-run evolvability enhanced by a certain transposon content in the host genome, illustrating how the different levels of selection are tightly connected in a complex interplay.

### Integration site preference of DNA transposon elements

Along with genomic excision transposons are faced with the task of locating new sites in the genome to integrate into. The target site preference of transposons varies between the different transposon families, but common to most elements is that the target site sequence is duplicated upon integration, leaving the inserted transposon flanked by small stretches of identical sequences. Some transposon elements are very strict in their sequence choice, such as the Tc1/*mariner* elements which always integrate into a TA dinucleotide [127], and *piggyBac* which always integrate into TTAA tetranucleotides [27]. Other transposon elements, such as the *hAT* superfamily and *P* elements, are more flexible and insert into 8-bp integration sequences that may vary in nucleotide composition [73,128].

In addition to the primary target site sequence, several genomic features influence transposon insertion site preference. One feature is the genomic distance from the transposon donor site. In a study of *Sleeping Beauty* transposition in the mouse germ line it was observed that 27% of the transposition events had occurred within 200 kb of the donor site, and 75% of the transposition events were found to be on the same chromosome [129]. This phenomenon, called local hopping, has been found for numerous other transposable elements such as the Tc1 element [130], *P* elements [131], the *Tol2* element [132], and *Ac/Ds* elements [133]. As *Sleeping Beauty* insertion sites are widely distributed in the genome, when transposition occurs from a donor plasmid [50,51], local hopping most likely reflects a tendency of the transposon to select target sites that are physically close to the donor site rather than a preference for some chromosome sequences over others. Interestingly, the *piggyBac* transposon seems to exhibit no or little local hopping [134,135], suggesting that this element has a different way of reaching its target site relative to the majority of DNA transposable elements.

Another genomic feature that has importance for insertion site preference of several Tc1/*mariner* elements is DNA bendability. Thus, the flexibility and overall topology may influence the access to the DNA at a given genomic position. Analyses of insertion sites of Tc1, Tc3, *Himar1*, *Sleepng Beauty*, and *Minos* transposons have shown that TA sites in DNA regions with increased bendability are preferred compared to TA sites in more stiff DNA regions [50,51,136,137]. The exact molecular mechanism governing this Tc1/*mariner* preference still remains to be elucidated, but a possible explanation could be that flexible DNA is more easily attacked by the transposase catalytic site during transposition, and/or that the transposase interacts with cellular proteins associated with bendable DNA. Based on studies demonstrating the sensitivity of *Hsmar1* transposition to DNA topology, it was speculated that a certain topology of the targeted sequence enables the transposase to create mechanical strain at the active site by bending the DNA. This may allow structural changes during release of the mechanical strain, making re-excision of the inserted transposon less likely [56].

Retroviruses have been shown to have a non-random integration profile with some genomic locations being favored over others. Lentiviruses, for instance, prefer to integrate into actively transcribed units, while gammaretroviruses have a preference for integrating near the 5′ end of actively transcribed genes [138]. Among the DNA cut-and-paste transposons, some elements appear to have a random integration profile while others have integration profiles that resemble those seen for retroviruses. Analyses of *Sleeping Beauty* insertion sites from cultured HeLa and K562 cells [50,139], mouse liver [51], NIH 3 T3 mouse fibroblasts [51], and human primary T cells [140], have revealed that the *Sleeping Beauty* transposon has a fairly random integration profile with no preference for or against genes. The *P* element, in contrast, has a strong preference for integrating near promoter regions, and analyses of more than 9,000 insertions in the *Drosophila* genome showed that 73% of *P* element insertions lie within 500 bp of an annotated 5′ transcription start site [141,142]. A recent study showed that promoters are not randomly targeted. Although 71% of 18,213 insertions analyzed were associated with promoters, only 24% of the total amount of annotated promoters in the *Drosophila* genome were targeted by an insertion [143]. Furthermore, a strong correlation was seen between *P* element target sites and Origin Recognition Complex (ORC) binding sites at replication origins. ORC associates with open chromatin and promoters thus explaining the correlation between *P* element targets and promoters [144]. Also the *Tol2* and *piggyBac* transposons are shown to exhibit nonrandom integration patterns. Of 113 *Tol2* insertion sites in HeLa cells, 48% were found to be in transcriptional units and a significant number was observed to be close to transcriptional start sites [145], a preference that was also seen for *Tol2* in HEK293 cells [146] and in primary human T cells [140]. Similarly, a bias towards transcriptional start sites and intragenic regions have been observed for the *piggyBac* transposon in HeLa and HEK293 cells [28,63,146], mouse embryonic stem cells [134], and primary human T cells [140,147]. In addition, *piggyBac* has been observed to have a preference for integrating into active genes in primary human T cells [147]. The preference of lentiviruses to insert into actively transcribed units has been associated with their binding to the ubiquitously expressed nuclear protein LEDGF/p75 (reviewed in [148]). LEDGF/p75 functions as a transcriptional coactivator, and the lentiviral integrase protein has been observed to bind to the C-terminus of LEDGF/p75, while the N-terminus of LEDGF/p75 can bind to chromatin, thereby tethering the viral integration complex to actively transcribed regions. Interactions between DNA binding proteins associated with transcriptional regions and DNA cut-and-paste transposons have not been observed so far, but perhaps such interactions accounts for the nonrandom integration profile seen for some transposable elements.

### Invasion, spread, and regulation in host genomes

In a likely scenario in nature, transposon colonization of the germline of a species is initialized by horizontal transmission between species followed by dramatic multiplication and spread of the mobile DNA element. Over time, the copy number will level out at a steady-state level as the result of the co-evolution of host and transposon. The delicate balance between continuous spread and proliferation of active elements and a decline in host fitness caused by transposon-induced damage to the genome results in selection at the host level against high transposon activity. It also seems reasonable that transposons on this leg of their evolutionary journey travel with regulatory mechanisms that may directly influence the transposition rate and reduce the genetic harm to the host. Although such control mechanisms are vaguely characterized, there is evidence that transposons not only are regulated by host defense pathways, including transcriptional silencing and posttranscriptional silencing by RNA interference (see later), but also that the elements themselves possess autoregulatory control mechanisms with the transposase protein as the key player.

### Transposon autoregulation by transposase overproduction inhibition

In attempts to mimic DNA transposition in nature most studies of DNA transposons with activity in eukaryotes rely on genetically engineered transposons and cloned versions of transposases that have been genetically modified to obtain activity. In addition, in functional studies transposase expression is often driven by promoters that facilitate high levels of transposase production. Despite potential discrepancies between actual mechanisms that are in play during genome evolution and in short-term laboratory assays, there is solid evidence that DNA transposition of members of the Tc1/*mariner* and other transposon superfamilies is inhibited by elevated levels of transposase, indicating, hence, that transposon activity during evolution has been regulated by self-inhibitory regulatory mechanisms. Events of transposase-based autoregulation, collectively referred to as overproduction inhibition (OPI), were first described for eukaryotic transposons by Lohe and Hartl [149], but had seemingly been observed in previous *in vitro* transposition studies including purified transposase protein derived from the mobile prokaryotic element *Tn10*[150]. In transposase activity studies based on germline excision of a target *mariner* transposon present in the genetically engineered *white-peach* gene of *Drosophila mauritiana*, Lohe and Hartl found reduced levels of mariner excision as a result of increasing transposase dosage. Hence, homozygous flies carrying two *Mos1* transposase-expressing alleles or flies carrying copies of the transposase expression cassette on two different chromosomes showed significantly lower excision rates than flies with a single cassette [149,151]. In addition, *Mos1* excision was further reduced by heat-shock treatment of flies containing transposase genes driven by a chimeric heat-shock *hsp70*-derived promoter, resulting in excision rates that were reduced more than 50% in comparison to untreated flies carrying a single transposase gene [149,152,153]. In potential contradiction to these findings, even though excess amounts of *Mos1* impairs paired-end complex assembly in a mobility shift assay, increasing dosages of purified *Mos1* transposase expressed in *Escherichia coli* were found not to cause OPI in an *in vitro* transposition assay [154,155]. In similar assays, purified transposase derived from the reconstructed *Himar1* mariner element was found to induce strong OPI even at low concentrations [57,154]. Transposition studies in HeLa cells and rabbit synovial fibroblasts confirm that increasing amounts of transfected *Himar1* transposase-expressing plasmid result in reduced transposition rates [58].

Despite recent evidence for activity of the *Himar1* element in human cells [58], the functional properties of the *Sleeping Beauty* transposase has attracted special attention due to its high activity in human cells [4]. By transiently expressing the *Sleeping Beauty* transposase from a heat-inducible promoter and from two strong constitutive promoters, Izsvak and coworkers were not able to detect any negative regulatory effects of high transposase production levels in cultured HeLa cells. Hence, within the studied levels of transposase (as detected by western blotting) the transposition rates did not plateau or decrease with increasing transposase expression. [39]. However, by co-transfecting plasmids containing the transposon substrate and the transposase expression cassette, respectively, in a broad range of ratios ranging from 1:17 to 33:1 in HeLa cells (keeping the total amount of DNA constant) dramatic inhibitory effects on transposition were observed with higher doses of transposase [156]. In these experiments, the highest level of transposition was obtained with a transfection mixture containing 0.5 μg transposon plasmid and 0.1 μg of a plasmid containing a CMV-driven transposase expression cassette, but was reduced 12-fold by including 10 times more transposase-encoding plasmid in the transfection mixture. In an identical transfection experiment, performed in HT1080 cells, the *Sleeping Beauty* transposition rate declined only moderately (about 1.5-fold) [157], indicating that *in vitro*-documented OPI effects of Tc1/mariner elements may be influenced by factors like cell type and transfection rate.

Based on many reports from the last years, it appears now to be generally accepted that *piggyBac*, and optimized derivatives of this system, may transpose in human cells with efficiencies that are comparable to improved versions of the *Sleeping Beauty* transposon. Although a single study shows OPI of *piggyBac* in HEK-293 cells [60], it appears that these non-Tc1/*mariner* transposases are generally less sensitive to OPI [60,63,157]. Indeed, in a direct comparison between the *Sleeping Beauty*, *piggyBac* and *Tol2*, the hyperactive SB100X transposase was shown to be more prone to regulation by OPI, reaching optimum transposition conditions at a 1:10 transposase:transposon ratio [145]. Notably, one report stated that molar *piggyBac* transposase-to-transposon ratios as high as 43:1 did not induce OPI in HEK-293 cells, whereas a similar ratio for *Sleeping Beauty* caused OPI [63].

OPI-based regulation of transposition represents an additional layer of complexity in transposon-based gene transfer and may have crucial influence on the *in vivo* use of transposable elements. However, the question remains if negative dosage effects observed in transient transfection studies reflect true biological mechanisms with impact on natural populations and the regulation of DNA transposition through evolution or, perhaps less excitingly, is the result of artificial overproduction of an enzyme with toxic effects on the treated cells. One can easily envisage putative evolutionary implications of OPI as one of many possible ways of regulating genomic transposition. In a likely scenario, low levels of transposase expressed from a relatively low number of integrated elements may provide optimal conditions for mobility. However, as the number of active elements increases over time the overall transposase dosage may reach a certain threshold beyond which transposition is reduced. As of today the mechanisms involved are unknown but may possibly include posttranslational interactions between transposase molecules. Recent studies of a resurrected *Hsmar1* mariner transposon support a model by which transposon activity is autoregulated through competition between transposase subunits for binding sites within existing transposon elements [158]. In accordance, it has been suggested that overproduction leads to transposase oligomerization and reduced transpositional activity [149]. In another plausible scenario, a surplus of free transposase molecules form complexes with molecules already bound to the transposon, thereby inhibiting interactions between the inverted repeats during transposition. This ‘quenching’ mechanism would imply most likely that an increase in the amount of transposon substrate in transfections should reduce the extent of OPI at a given level of transposase. However, in at least one *Sleeping Beauty* study this was found not to be the case [156].

### Regulation and silencing by epigenetic modifications

Eukaryotic genomes consist of regions of transcriptionally active euchromatin and transcriptionally inactive heterochromatin. The organization of genomes into such regions is controlled by an interplay between DNA methylation and histone modifications, and these epigenetic modifications have been shown to be closely associated with the transcriptional state of many eukaryotic genes [159,160].

Analyses of DNA methylation patterns in both plant and animal species have revealed that endogenous transposon elements are heavily methylated [161]. Recent genome-wide DNA methylation analyses confirm that this is the case also in human embryonic cells and somatic tissues [162]. Such DNA methylation may correlate with silencing of mobile DNA elements, as increased mobilization of transposons in both plant and animal mutants is linked to abolished DNA methylation. The findings that silent transposable elements can be reactivated upon lack of DNA methylation has led to the belief that epigenetic modifications and heterochromatin formation represent basic defense mechanisms to prevent the harmful activity of mobile DNA elements within genomes [163]. Notably, recent evidence suggests that a minor portion of human transposable elements is hypomethylated in a tissue-specific manner, supporting a model by which transposon elements may possess enhancer-like functions and assist in regulating genes [162].

Studies of transposition in *Arabidopsis thaliana* mutated in the *DDM1* (decrease in DNA methylation) gene revealed increased transposition rates [164,165]. Similar results were observed by Lippman *et al.* who, by microarray and chromatin immunoprecipitation (ChIP) studies in *A. thaliana*, showed that heterochromatic CACTA transposons and gypsy-like retrotransposons are activated in *DDM1* gene mutants [166]. These results, however, could also be explained by the implication of DDM1 in chromatin remodelling [167]. Nevertheless, in a later study Kato and colleagues showed that transposition of CACTA elements in *A. thaliana* was enhanced significantly in plants defective in either CG or non-CG DNA methylation[168]. Although transposition assays on hemi-methylated DNA are needed to give firm support from other superfamilies, these studies on CACTA and Mutator indicate profound effects of DNA methylation on transposition – perhaps caused by an underlying replication mechanism based on methylation-state-regulated transposition. A correlation between decreased cytosine methylation and increased transposition of *IAP* retrotransposons was also observed in mouse embryos deficient of a fully functional *Dnmt1* (DNA methyltransferase-1) gene [163].

As an intriguing exception to these findings, it appears that *Sleeping Beauty* transposition is enhanced by transposon DNA methylation, suggesting that transposon mobilization is supported by CpG methylation [169]. Recent findings have confirmed such supportive role of methylation for mobilization of *Sleeping Beauty* and *Frog Prince* elements, leading to the assumption that methylation and a resulting tight chromatin structure is beneficial for transposition of these particular elements [170]. In mouse embryonic stem cell lines, containing a single *Sleeping Beauty* transposon insertion, excision efficiencies were observed to be 100-fold higher when the *Sleeping Beauty* transposon was in a heterochromatic conformation compared to control clones without the heterochromatic conformation [171]. Improved transposition efficiency of a methylated *Sleeping Beauty* transposon in mouse embryonic stem cells was also observed in a study in which transposition efficiency of the *piggyBac* transposon at the same time was shown to be reduced upon methylation [134]. In accordance, immunostaining and biochemical analyses have shown that the *Sleeping Beauty* element appears to have an affinity for heterochromatic regions [171]. It is tempting to propose that such mechanism in an evolutionary context would facilitate escape of the transposon from heterochromatic and methylated regions of the genome, but explanations for this observation remain elusive.

The *Tol1* and *Tol2* transposable elements, both members of the *hAT* superfamily, are unique among the DNA transposons, as they are the only natural active elements to have been discovered so far in vertebrate genomes. The *Tol2* element has had a rapid expansion in the genome of its host, the medaka fish, in the past, but a high spontaneous transposition rate is not observed in the genome of current laboratory fish strains, suggesting that *Tol2* has already reached a steady-state level where the transposition frequency is controlled by host mechanisms [172]. Evidence links DNA methylation to host control of *Tol2* transposition. Hence, in a study by Iida *et al*. [173] medaka fish embryos were soaked in 5-azacytidine, a reagent that acts as a false substrate and potent inhibitor of methyltransferases leading to reduction in DNA methylation. Reduced CpG methylation levels together with increased transposition excision frequencies were observed in the treated fish embryos. Interestingly, the 5-azacytidine treatment did not seem to induce increased expression of the transposase gene, suggesting that DNA methylation of the *Tol2* transposon did not inhibit transposition by transcriptional silencing of the transposase gene. In studies involving other *hAT* elements, methylation at the ends of the plant transposon *Tam3* (from *Antirrhinum*) was shown to cause repression of transposition [174], and DNA methylation at the transposase binding sites of the *Ac/Ds* element was shown to inhibit *Ac* transposase binding [95]. These findings support the notion that transpositional regulation by DNA methylation in the *hAT* transposon superfamily is due to inhibited transposase binding to transposon binding sites.

Rather than affecting the process of transposition itself, epigenetic modifications may impact transposase expression and thereby indirectly regulate transposition activity. CpG methylation of the *Sleeping Beauty* element carrying a transgene, and not the natural transposase gene, was analyzed in transgenic mice containing single-copy transposon insertions [175]. The DNA methylation status of the inserted transgene cassette, which consisted of the ROSA26 promoter and the eGFP gene, was examined by bisulfate-mediated sequencing for six independent insertions. The results showed that *Sleeping Beauty* transposons inserted into the mouse genome were heavily methylated in the ROSA26 promoter and the eGFP coding sequence. In contrast, the endogenous mouse ROSA26 promoter was devoid of CpG methylation in the transgenic mice. It appears that the host cells were able to distinguish between the endogenous genomic sequence and the exogenous counterpart inserted by *Sleeping Beauty*, suggesting that the transposon was specifically recognized and targeted by the host cellular epigenetic modification system. In another study, however, *Sleeping Beauty* transgenic mice containing a transgene cassette comprised of a human K14 promoter and the Agouti reporter gene did not show significant levels of CpG methylation at the inserted transgene [176], indicating that *Sleeping Beauty* cargo DNA sequences rather than transposon inverted repeat sequences influence CpG methylation status. We have at several occasions observed transcriptional down-regulation and silencing of *Sleeping Beauty*-inserted transgene cassettes [177-179], but the promoter rather than the carrier seems to a key determinant for such silencing. Nevertheless, in comparative studies performed in human retinal pigment epithelium cells, a transgene cassette delivered by *piggyBac* was less vulnerable to silencing than a cassette delivered by the *Sleeping Beauty* counterpart, lending support to the notion that the level of silencing was influenced by the distinct integration profiles [179].

The role of histone modifications in transposon control is under continuous investigation. A recent study in murine embryos showed reactivation of the *LINE-1* and *IAP* retrotransposons in 2-cell embryos. A subsequent loss of expression in 8-cell embryos was shown to correlate with a loss of the activating trimethylation of H3K4 rather than gain of the repressive trimethylation of H3K9 [180]. It is not currently known to which degree DNA transposition is directly affected by histone modifications.

### Regulation by RNA interference (RNAi)

**Figure 5:**
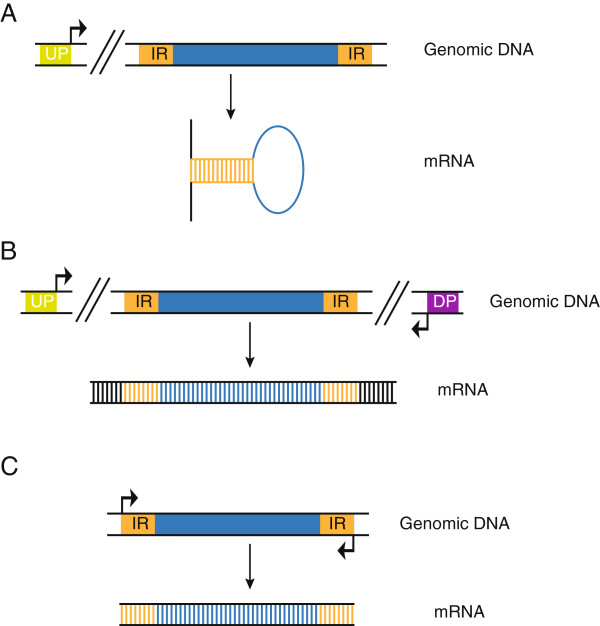
**Transposon regulation by RNA interference. (A)** An upstream promoter (UP) generates read-through transcripts that fold back on themselves due to intramolecular base pairing of the inverted repeats (IRs), thereby generating dsRNA transcripts that may be processed into RNAi effectors. **(B)** Upstream and downstream promoters (DP) create bi-directional RNA transcripts that anneal and form dsRNA. **(C)** Promoter-like activity in the inverted repeats generates sense and antisense transcripts which, as in **(B)** anneal to form dsRNA.

To produce transposase proteins that facilitate jumping of transposons to a new location in the genome the transposon open reading frame must first be copied into mRNA. The evolution of host silencing mechanisms, based on the ability of small inhibitory RNA molecules to guide mRNA degradation in a sequence-specific fashion, has allowed higher organisms to defend the genome against mobile elements and harmful effects of insertional mutagenesis and genomic instability. Formation of transposon-derived double-stranded RNA (dsRNA) is a hallmark of RNAi-based suppression of transposition. Figure 5 provides a schematic representation of three potential scenarious for production of transposon-derived dsRNA that may be processed into effectors of a regulatory RNAi response. These include intramolecular basepairing between terminal inverted repeats of read-through RNA transcripts (Figure 5A), basepairing between bidirectional RNA transcripts generated from sense and antisense promoters within the element (Figure 5B), or intermolecular annealing of sense and antisense read-through transcripts (Figure 5C).

The lack of easy identifiable regulatory elements in DNA transposons has fueled the idea that expression of the transposase gene in original elements depends on endogenous regulatory sequences flanking the inserted transposon. This may give rise to read-through RNA transcripts that may serve as templates for transposase synthesis. In *C. elegans*, transcription of *Tc1* sequences is initiated in the flanking sequences and not within the inverted repeats [181]. Interestingly, such read-through transcripts may form dsRNA by folding back on themselves, allowing complementary intramolecular inverted repeat sequences to anneal (Figure 5A). These dsRNA regions are processed by the cellular RNAi machinery, triggering silencing of the transposable element by transposon-specific RNA degradation [181]. As *Tc1* transposition in wildtype worms occurs only in somatic cells and not in the germ line, identification of mutants with active transposition in germ cells has led to identification of genes that play an active role in the RNAi response in *C. elegans*[182,183]. With our current knowledge, it is not surprising that these genes, *rde-2* and *mut-7* among others, encode conserved proteins involved in degrading RNA by RNAi. In agreement with data showing that mutations in these genes relieve a block on transposition [182,183], small RNA effectors of the RNAi machinery (referred to as small interfering RNAs, siRNAs) derived from *Tc1* are much less abundant in mutant worms defective in both RNAi and transposon silencing [181].

Fastly accumulating evidence suggests that retrotransposons in wide variety of host organisms are suppressed through the RNAi pathway. In mouse oocytes, repeat-associated siRNAs (ra-siRNAs) derived from retroelements have been identified and are likely effectors in an anti-transposon response through RNAi [184], as supported by findings showing high abundance of retrotransposon-derived transcripts in Dicer-deficient mouse embryonic stem cells [185]. Moreover, RNAi-based Dicer knockdown in preimplantation mouse embryos causes an increase in abundance of transcripts from LTR retrotransposons [186]. In the *Drosophila* germline, ra-siRNA-directed silencing serves to suppress transposon expression, but through a yet incompletely understood RNA processing pathway that does not involve siRNA production by Dicer. Such ra-siRNA effectors are referred to also as piRNAs due to their association with the Argonaute subfamily of PIWI proteins [187,188] and have been found to suppress activities of both retrotransposons and DNA transposons in the zebrafish and mouse germline [189,190]. Intriguingly, PIWI/piRNA complexes not only exercise their function on a post-transcriptional level, but have recently been shown to enforce transcriptional repression of transposable elements [191,192] and to recruit HP1 to confer repressive chromatin marks when bound to euchromatin [193]. Furthermore, a loss of repressive marks at transposon loci is shown in *Drosophila* PIWI mutants.

*LINE-1* retroelements comprise about 17% of the human genome and an estimated 100 of these elements are fully capable of transposition. Sense and antisense promoters in the 5′ untranslated region of *LINE-1* direct production of bi-directional RNA transcripts which can potentially anneal to form double-stranded RNA (Figure 5B). Such dsRNA molecules are processed to siRNAs which can suppress *LINE-1* retrotransposition in human cells [194,195]. Notably, loss of the antisense promoter is accompanied by increased *LINE-1* transposition, and knockdown of Dicer by synthetic siRNAs causes an increased abundance of endogenous *LINE-1* RNA transcripts in cultured human cells [194], both findings suggesting that *LINE-1* retrotransposition is suppressed by RNAi.

We previously showed that the terminal IR sequences of the *Sleeping Beauty* DNA transposon possess moderate gene-regulatory activities [196]. IRs oriented against the center of the transposon stimulate gene expression in transient reporter assays and have a considerable impact on the expression of genes within the integrated transposon. We therefore envisage a scenario reminiscent of RNAi suppression of *LINE-1* elements and speculate that opposing transcriptional activities driven by the element itself, or neighbouring regions, may influence transposition by mechanisms that may possibly involve transcriptional interference pathways. These findings suggest that DNA transposable elements during evolution in higher vertebrates have been held in check partially by RNAi-related pathways. This notion is supported by the fact that *Sleeping Beauty* DNA transposition is enhanced in cells in which the RNAi machinery is suppressed by expression of the P19 protein [197]. It is unclear yet, however, whether the *Sleeping Beauty* IRs serve as primitive promoters or support gene expression by other mechanisms. Interestingly, similar regulatory activities of the *C. elegans Tc3* element [196] could indicate that bidirectional transcription of DNA transposons is a conserved feature of DNA transposable elements. However, elements with shorter inverted repeats, e.g. *Tc1*, may rely on read-through transcription (Figure 5C), as suggested by the finding that *Tc1* inverted repeats do not possess promoter-like activities [196].

### Ending the journey – dominant-negative complementation and transposase titration

**Figure 6:**
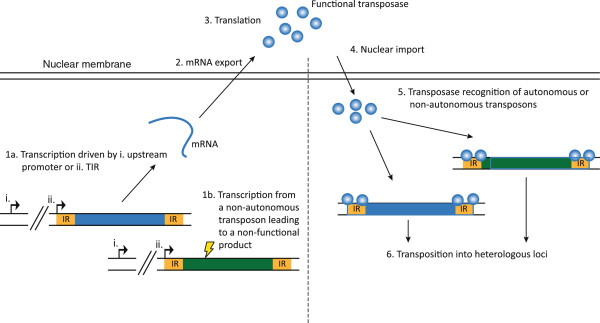
**Mobilization of autonomous and non-autonomous transposons.** Transcription from autonomous transposons is driven by either an upstream promoter **(i)** or by the IR **(ii) (1a)**. Non-autonomous transposons comprising loss-of-function mutations (indicated by lightning bolt) are unable to make a functional transposase **(1b)**. mRNA from the autonomous transposon is exported to the cytoplasm **(2)**, translated into functional transposase **(3)**, which is transported into the nucleus **(4)**. The transposase recognizes both autonomous and non-autonomous transposons **(5)** leading to transposition of both **(6)**.

Transposable elements cannot keep racing evolution forever and eventually run out of gas, as evidenced by the numerous DNA transposons that reside as inactive genetic relics in genomes throughout the animal kingdom. The mechanisms underlying this fate have been addressed mainly for the *P* element and Tc1/*mariner* superfamilies, but likely apply to all cut-and-paste DNA transposons. The probability of further spreading of transposable elements and the inherent risk of insertional mutagenesis is reduced over time due to the accumulation of loss-of-function mutations in the transposase gene. While such mutations will be favored by selection at the host level, they will be nearly neutral at the transposon level, as the transposase proteins produced by intact (autonomous) elements cannot distinguish autonomous from non-autonomous elements in choosing its substrate (Figure 6). Mutations will thus over time accumulate in the transposase genes, rendering more and more transposons non-autonomous, a process referred to as ‘vertical inactivation’ [79]. Besides the direct effect on transposition activity by lowering the amount of functional transposase, vertical inactivation promotes at least two indirect mechanisms of transposition regulation. Firstly, inactive transposase protein is thought to interact with active transposase protein to inhibit transposition in a process termed dominant-negative complementation (DNC) [149]. Secondly, the non-autonomous transposons produced by vertical inactivation might still serve as substrate for active transposase, hitching free rides at the cost of autonomous transposons. By this mechanism, called transposase titration [198], the proliferation rate of autonomous elements and, hence, further transposase production is hampered. Vertical inactivation thus continuously lowers transposition activity until all elements present in the genome are inactive. Without transposition activity or selection the population of inactive transposons will slowly be lost from the genome by genetic drift (stochastic loss [79]). At this point all hope is out for the paralyzed and dying transposons. To survive as a group of mobile genetic elements horizontal transfer must occur before all elements are inactivated, as proliferation and maintained transposition activity is required to colonize naive host genomes.

### Mobile DNA elements as therapeutic gene vehicles

Since its discovery in 1997 the *Sleeping Beauty* transposon has been extensively studied for genetic applications in vertebrates. As we have learned more about the biology of *Sleeping Beauty* and its advantages and potential drawbacks as a vector for therapeutic gene delivery, several alternative DNA transposons with activities in human cells have appeared on the scene. Although active in human cells, elements like *Tc1* and *Tc3* from *C. elegans*, *Mos1* and *Minos* from *Drosophila*, and *Himar1* from hornfly do not appear to transpose with the activity of *Sleeping Beauty* in human cells [60,170,199] and therefore have not attracted much attention as therapeutically relevant gene vehicles. In contrast, elements like *piggyBac*, *Tol2*, *Passport*, and *Frog Prince* have shown robust transposition in human cells [29,30,33,60,63,200] and may serve, therefore, as attractive alternatives to *Sleeping Beauty*-derived gene vectors. Much of the focus, however, has been put on the *piggyBac* element as an alternative to *Sleeping Beauty* in mediating therapeutic gene insertion. The therapeutic properties of both *Sleeping Beauty* and *piggyBac* systems are covered in several excellent reviews [44-46,201].

Common to these mobile elements is that they - during invasion, genomic spread and regulation - have been shaped by an evolutionary drive for survival and maintenance of high copy numbers in their natural hosts. As a consequence, differences between the systems and their performance in human cells may likely be influenced by how far they have reached on their evolutionary journey. Hence, it can be hypothesized that invading autonomous elements have high activity but also may not yet have adapted to regulatory defense mechanisms of the host. In addition, such elements may not yet have evolved self-regulatory mechanisms which are likely to support a steady-state level of transposition and a balanced number of elements after initial invasion. In any circumstance, each of the isolated elements is the result of a genetic interplay between element and host, and the biological properties are therefore more than likely to vary between the elements. With current interests in employing DNA transposons for gene delivery it is important to make the point that the vehicles, which we create for gene transfer purposes in humans, are derived from elements formed by an ongoing evolutionary journey and therefore may carry different functional properties as result of variable selective pressures. This stresses the necessity of creating vectors based on different mobile elements and, moreover, fully characterizing the biological properties of such vectors. Except for *Tol2* and *piggyBac*, which both encode a functional transposase and are still active in their original host genome, current elements with high activity in human cells originate from fossils which have accumulated mutations, rendering them inactive in their natural hosts. As reawakening of vertically inactivated elements relies on a human touch of site-directed mutagenesis, it is likely also that differences between the systems may originate, at least partially, from different strategies and results of reconstruction. Moreover, it can be argued that elements with a history in vertebrates, like *Tol2* and *Sleeping Beauty*, may have evolved under cellular conditions that may favor their optimized use in human cells as opposed to elements derived from insects or nematodes.

Despite the proven potency of *Sleeping Beauty*-mediated gene insertion for a range of applications in various tissues, *Sleeping Beauty*-based vectors do indeed carry traits of their heritage and evolution that may represent potential shortcomings in a therapeutic scenario. Phylogenetic analyses have suggested that *Sleeping Beauty* ancestors have evolved through reassortment of functional domains between mobile elements and horizontal transmission [202]. Hence, members of *Tc1*-like transposon family are prevalent in many fish genomes but are inactive due to either (i) accumulation of mutations in the transposase gene or (ii) more severe genetic alterations, like deletions, caused by vertical inactivation. Among *Tc1*-like elements in fish the salmonid subfamily appears to be the youngest and most recently active [202]. Based on phylogenetics of this subfamily the pioneering reconstruction work performed by Zoltan Ivics and Zsuzsanna Izsvák in the laboratory of Perry Hackett brought a functional ‘consensus’ element, perhaps identical but more likely equivalent to an riginal ancient element, back to life. Since the initial discovery, hyperactive variants of the *Sleeping Beauty* transposase have been developed by mutagenesis, leading to several versions of the transposase with enhanced transposition properties [52,136,203]. Ultimatively, a high-throughput PCR-based DNA-shuffling strategy was utilized to produce the hyperactive transposase SB100X, which was found to be 100-fold more active than the original *Sleeping Beauty* transposase (originally designated SB10) under certain experimental conditions [204]. Moreover, higher levels of *piggyBac* transposition have been achieved by codon optimization of the transposase gene, leading to induced levels of expression [205,206]. Also, a hyperactive *piggyBac* transposase (HyPBase) was recently identified by screening of a transposase mutant library and combining beneficial mutations in a single transposase variant [64]. By employing mouse liver as an *in vivo* model, SB100X and HyPBase have been found to increase *in vivo* efficacy above what has been observed with previous transposase variants [29,53] and currently represent the first choices for therapeutic transposition of genes. Although such variants may prove to be prominent tools for gene insertion in hard-to-transfect cell types or tissues, it should be noted that such transposases with an enhanced gene-inserting potential may also pose an increased risk of causing insertional mutagenesis.

### Delivery of transposons and transposases

**Figure 7:**
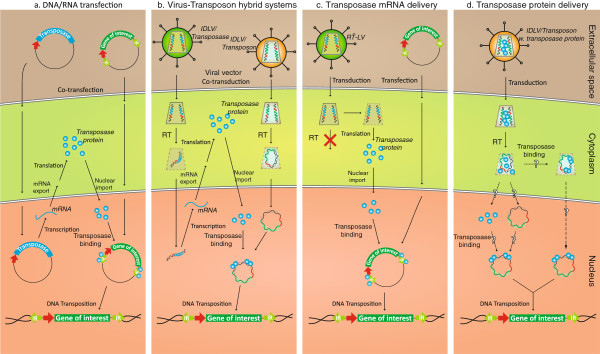
**Schematic representation of four different approaches for delivery of components of the DNA transposon-based gene delivery systems. Panel a** illustrates the conventional delivery of transposase and transposon by plasmid DNA transfection. This approach relies on nuclear uptake of both helper and donor plasmid DNA allowing transcription of transposase-encoding mRNA, mRNA export, production of transposase in the cytoplasm, and subsequent nuclear import of transposases. Transposases bind to the transposon donor plasmid and facilitates transposition. A variant of this approach is based on transfection of *in vitro*-transcribed mRNA encoding the transposase (not shown). **Panel b** represents an emerging approach based on virus-mediated delivery of DNA transposon systems. The example shown demonstrates the use of integrase-defective lentiviral vector (IDLVs) as carriers of the transposase gene (left) and the transposon (right), allowing transposition from reverse-transcribed (RT) lentiviral DNA intermediates (here represented by circular forms). Related approaches have been developed for vectors based on adenoviruses, adeno-associated viruses, and herpes simplex viruses. **Panel c** illustrates the use of reverse transcription-defective retroviral vectors as carriers of transposase-encoding mRNA. Modifications of the primer binding site, where reverse transcriptions is normally initiated by an annealed tRNA, inhibit reverse transcription and thus facilitating vector RNA delivery, and direct translation into protein. The transposon donor is in this example delivered by plasmid DNA transfection. **Panel d** demonstrates the possibility of delivering DNA transposon systems in engineered ‘all-in-one’ lentiviral particles that co-deliver both transposase protein and the donor for DNA transposition. Transposase subunits delivered by lentiviral protein transduction are delivered in the virus context and facilitate efficient transposition through mechanisms that may benefit from the close interaction between transposases and the reverse-trancribed donor within the viral pre-integration complex. Question marks indicate that it is currently unknown at which stage transposases bind to the donor to form the synaptic transposition complex.

Conventional DNA transposon systems consist of two plasmids, one helper plasmid carrying the transposase expression cassette and one donor plasmid carrying the DNA transposon (Figure 7, panel a). After transfection, both plasmids find way to the nucleus, allowing production of transposase-encoding RNA from the helper plasmid and subsequent excision of the transposon from the donor plasmid facilitated by transposase subunits imported into the nucleus. This approach can be further refined by placing both the transposase gene and the transposon on a single plasmid, originally referred to as helper-independent transposon-transposase vectors [7]. Such one-plasmid systems do however require a stringent design to avoid OPI. Alternatively, transfected *in vitro*-transcribed mRNA may serve as a rich source of transposase [207], eliminating the risk of creating cells with prolonged expression of the transposase.

Alternative means of delivering the components needed in transposon-based gene delivery systems have emerged over the years. In prominent hybrid delivery systems the components of transposon-based vectors are carried and delivered by viral capsids, providing otherwise episomal vectors – like adenoviral [208-210] or herpes simplex virus-based vectors [211] – the ability to integrate genes and establish long-term transgene expression. Combining the best of two worlds the viral coat provides vector stability, tissue-specific transposon delivery and transport across the cellular membrane, while transposons facilitate viral vector integration according to the characteristic integration profile of transposable elements [51,208]. Viral vector-based transfer of the *Sleeping Beauty* transposon system was first demonstrated in mouse liver with adenoviral vectors [208], and recent studies have demonstrated the applicability of this approach in larger animals [210]. Also, adeno-associated viral vectors have been adapted as carriers of the *Sleeping Beauty* system [212]. We and others have established *Sleeping Beauty* DNA transposition from integrase-defective lentiviral vectors (IDLVs) (Figure 7, panel b), providing a new viral platform for delivering components of the *Sleeping Beauty* transposition system and altering the integration profile of lentivirally delivered transgenes [139,204,213]. Along these lines, retroviral vectors without the ability to undergo reverse transcription have been explored as a source of transposase-encoding mRNA (Figure 7, panel c) [214]. Most recently, we developed lentiviral protein transduction for direct delivery of transposase protein, allowing efficient DNA transposition in lentivirally transduced cells (Figure 7, panel d) [215]. Efforts to combine viral gene delivery with non-viral integration systems in ‘hybrid’ vectors have recently been reviewed [209,216,217].

### Preclinical evidence of transposon-based gene delivery

Among the elements with activity in mammalian cells *Sleeping Beauty* is by far the most studied for gene transfer purposes. Several studies have demonstrated high levels of gene insertion in mouse liver after hydrodynamic injection of naked DNA into the tail vein. Proof-of-principle was initially provided by Yant *et al.* who obtained persistent levels of human coagulation factor IX (hFIX) after hydrodynamic injection of plasmids carrying the transgene-tagged transposon and the transposase expression cassette, respectively [5]. Delivery of the SB100X transposase variant and hyperactive piggyBac was found later to provide even higher levels of transposition in mouse liver [53]. As this type of treatment was found to be therapeutic in a mouse model of hemophilia B [5], recent findings have demonstrated long-term phenotypic correction in immunotolerized hemophilia A mice treated with a transposon encoding human factor VIII (hFVIII) [8]. In addition, the *Sleeping Beauty* system has been successful in liver-directed treatment of mucopolysaccharidosis types I and VII [218] and tyrosinemia type I [219], and administration of *in vitro* synthesized RNA encoding the *Sleeping Beauty* transposase, as opposed to plasmid DNA, is sufficient to catalyze vector transposition in mouse liver [207,220]. Initial studies of *Sleeping Beauty*-directed transfer of hFIX showed efficacy but also demonstrated severe OPI in mice treated with too high levels of plasmid DNA with transposase expression driven by a standard cytomegalovirus (CMV) promoter. Hence, whereas a single microgram of transposase-encoding plasmid DNA was found to facilitate high levels of transposition, resulting in correction of the disease in mouse model of hemophilia, injection of 25 μg transposase-encoding plasmid DNA on the other hand did not result in DNA transposition [5]. In subsequent studies using helper-independent transposon-transposase vectors, the transposase expression was balanced by the use of a promoter that were markedly weaker than CMV [7]. It is not clear whether such OPI in mouse liver reflects true transposase-regulating mechanisms that mirror natural regulatory mechanisms of *Sleeping Beauty*, or rather is caused by toxicity in hepatocytes with high expression of the transposase. In either case, these findings marked the importance of recognizing specific characteristics of the vector technology and balancing the expression of the transposase for each specific application.

By administration of DNA-polyethylenimine complexes, *Sleeping Beauty*-containing plasmid vectors have been delivered to lung epithelial cells [12], allowing phenotypic correction in hemophilia A mice neonatally injected with a transposon vector encoding the hFVIII gene [16]. Gene expression in the alveolar region of *Sleeping Beauty*-treated mouse lungs may last as long as 3 months after injection [11]. Promising preclinical effects of *Sleeping Beauty* have been elegantly demonstrated in human glioblastoma xenografts in mice in which tumor-induced angiogenesis was inhibited by *Sleeping Beauty*-based co-expression of soluble vascular endothelial growth factor and a angiostatin-endostatin fusion variant [18,19]. Sustained tumor regression of intracranial gliomas was achieved only when functional *Sleeping Beauty* transposase was present, allowing persistent expression of the two genes.

## Conclusion

### Mobile elements heading for the clinic

Fifteen years have gone since the *Sleeping Beauty* element was re-awakened and soon after adapted for *in vivo* gene delivery facilitating persistent gene expression. Despite the various examples of pre-clinical efficacy for DNA transposon-based *in vivo* gene therapy, the road to the clinic will wind through additional experimentation and evidence of therapeutic effects in large animal models. As we keep learning more about the properties and details of the different transposable elements and continue developing both nonviral and viral delivery technologies, *in vivo* applicability could be waiting around the next corner. Until then, the history of mobile elements is helping us refining these engineering tools even further for use in both biomedical experimentation and clinical settings. With the current primary focus on the *Sleeping Beauty* and *piggyBac* elements, mechanisms that guide the distinct integration patterns, OPI, gene cargo capacities, putative cell type variations, and sensitivity to epigenetic silencing of these two elements seem to be key focus points for optimizing the performance of these elements further.

The combination of improved transposons, possibly mobilized from DNA minicircles free of bacterial sequences [221], and engineered hyperactive transposase variants has immediate potential in developing *ex vivo* therapies. Transposon-based gene insertion in plasmid-transfected primary T cells [222-225], hematopoietic stem cells [204,226-228], and human embryonic stem cells [229,230] is creating optimism for the use of DNA transposons in *ex vivo* genetic treatment. In fact, the first clinical trial based on transposon-directed gene integration aims at inserting a gene encoding a chimeric antigen receptor specific for CD19 in primary T cells [222] and has recently been initiated for treatment of patients with B-lymphoid malignancies with adoptive immunotherapy [231-233]. Through scenes of an evolutionary drive, DNA transposon-based vectors arrived at the clinic, but this is certainly not the final stop.

## Competing interests

The authors declare that they have no competing interests.

## Authors’ contributions

The manuscript was prepared by KAS, PRA, NS and JGM. All authors read and approved the final manuscript.
